# Heberden’s Commentaries

**Published:** 1845-11

**Authors:** 


### HEBERDEN'S COMMENTARIES.

This medical classic composes the October number of *Bell's Select Medical Library*, and will be welcomed by every reader. Heberden is a name familiar to every one versed in medical literature. His Commentaries belong to a former era in our profession, although he lived to see the opening of the present century, and consist, for the most part, of notes "taken in the chambers of the sick from themselves." The following are the beautiful words in which his observations are introduced to the reader.

"Plutarch says, that the life of a vestal virgin was divided into three portions; in the first of which she learned the duties of her profession, in the second she practised them, and in the third she taught them to others. This is no bad model for the life of a physician: and as I have now passed through the two first of these times, I am willing to employ the remainder of my days in teaching what I know to any of my sons who may choose the profession of physic; and to him I desire that these papers may be given."

He lived till he was nearly ninety-one years old, and his life and character are a model for the young physician. In the short biographical notice prefixed to the volume it is related that—

"From his early youth he had always entertained a deep sense of religion, a consummate love of virtue, an ardent thirst after knowledge, and an earnest desire to promote the welfare and happiness of all mankind. By these qualities accompanied with great sweetness of manners, he acquired the love and esteem of all good men in a degree which perhaps very few have experienced; and after passing an active life with uniform testimony of a good conscience, he became an eminent example of its influence, in the cheerfulness and serenity of his age." Y.

